# Supplementary material for: Assessing the Interactions of Statins with Human Adenylate Kinase Isoenzyme 1: Fluorescence and Enzyme Kinetic Studies
Source: Int J Mol Sci. 2021 May 24;22(11):5541. doi: 10.3390/ijms22115541 (PMC8197361; doi:10.3390/ijms22115541)
Supplement: Supplementary file 1 [file ijms-22-05541-s001.zip › ijms-1226165-supplementary.pdf]

International Journal of *Molecular Sciences*

MDPI

Supplementary data

Assessing the interactions of statins with human adenylate kinase isoenzyme 1: fluorescence and enzyme kinetic studies

Magdalena Wujak[1,2,†], Anna Kozakiewicz[3,*,†], Anna Ciarkowska[2], Joanna I. Loch[4], Magdalena Barwiolek[3], Zuzanna Sokolowska[3], Marcin Budny[5], Andrzej Wojtczak[3]

[1] Faculty of Pharmacy, Nicolaus Copernicus University in Toruń, Collegium Medicum in Bydgoszcz, Jurasza 2, 85-089 Bydgoszcz, Poland; mwujak@cm.umk.pl (M.W.)
[2] Faculty of Biological and Veterinary Sciences, Nicolaus Copernicus University in Toruń, Lwowska 1, 87-100 Toruń, Poland; anciar@umk.pl (A.C.)
[3] Faculty of Chemistry, Nicolaus Copernicus University in Toruń, Gagarina 7, 87-100 Toruń, Poland; akoza@umk.pl (A.K.), awojt@umk.pl (A.W.), mbarwiolek@umk.pl (M.B1.), z.a.sokolowska@gmail.com (Z.S.)
[4] Faculty of Chemistry, agiellonian University, Gronostajowa 2, 30-387 Kraków, Poland; loch@chemia.uj.edu.pl (J.I.L)
[5] Synthex Technologies Sp. z o.o., Gagarina 7/134B, 87-100 Toruń, Poland; budny@synthex.com.pl (M.B2.)
* Correspondence: akoza@umk.pl; Tel.: +48 56-611-4511
† These authors contributed equally to this work.

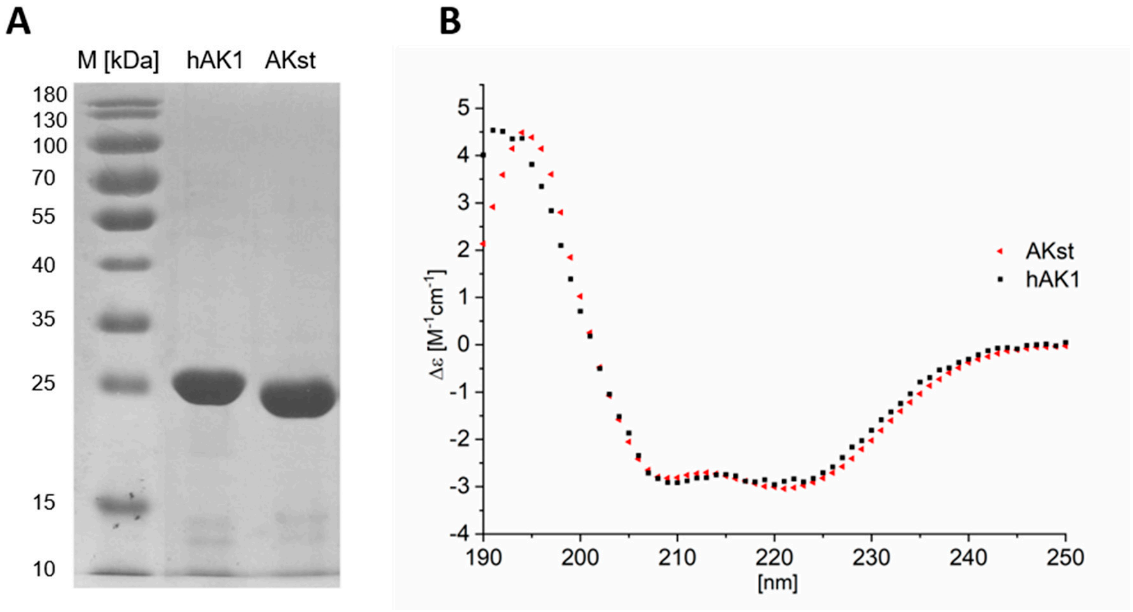

Figure S1. (A) SDS-PAGE and (B) Far-UV CD spectrum analysis of the purified adenylate kinases.

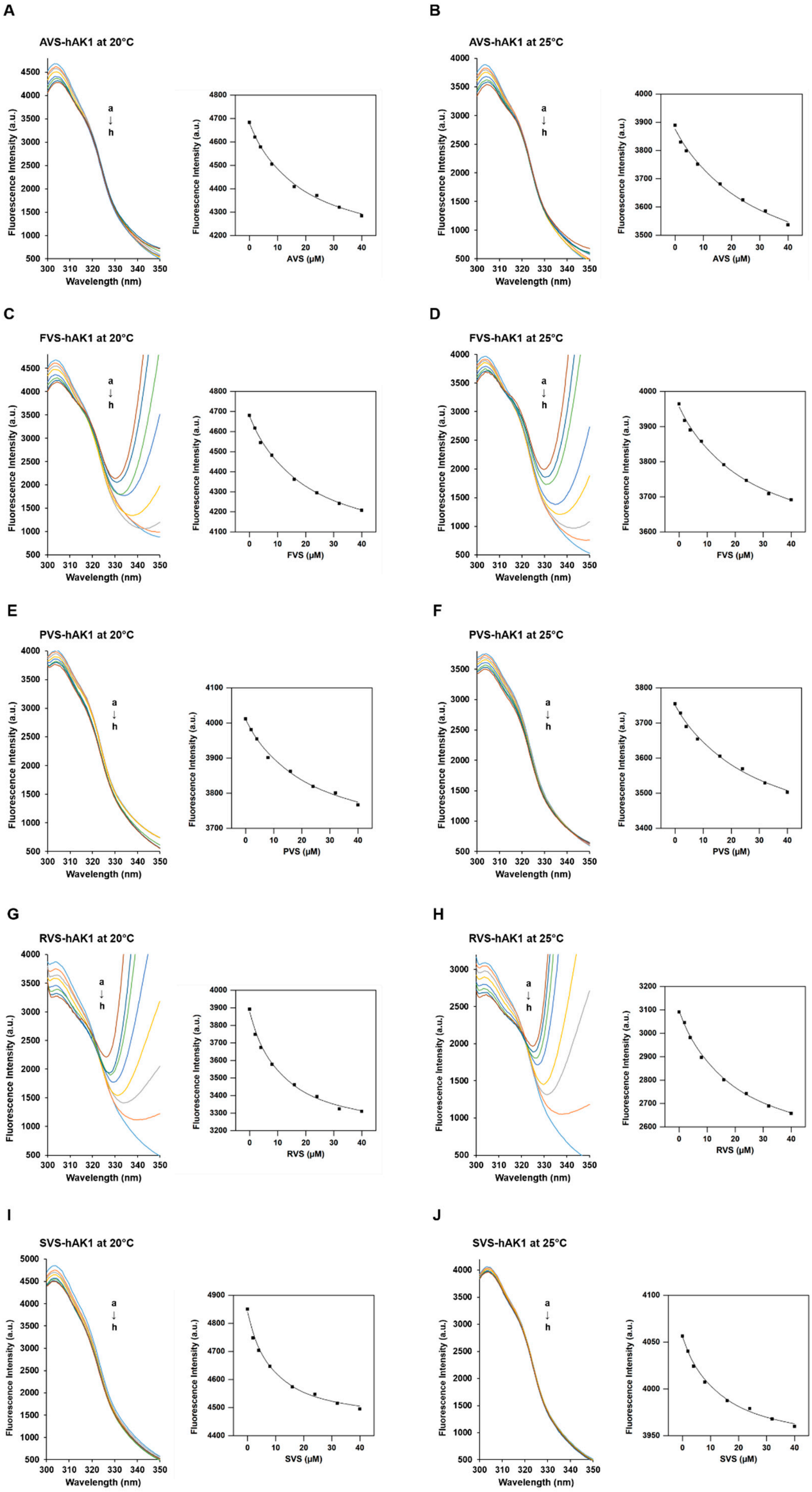
A
AVS-hAK1 at 20°C
B
AVS-hAK1 at 25°C
C
FVS-hAK1 at 20°C
D
FVS-hAK1 at 25°C
E
PVS-hAK1 at 20°C
F
PVS-hAK1 at 25°C
G
RVS-hAK1 at 20°C
H
RVS-hAK1 at 25°C
I
SVS-hAK1 at 20°C
J
SVS-hAK1 at 25°C
a
↓
h
Fluorescence Intensity (a.u.)
Wavelength (nm)
AVS (μM)
FVS (μM)
PVS (μM)
RVS (μM)
SVS (μM)

Figure S2. The fluorescence quenching of hAK1 in the presence of (A, B) atorvastatin, (C, D) fluvastatin, (E, F) pravastatin, (G, H) rosuvastatin and (I, J) simvastatin at (A, C, E, G, I) 20°C and (B, D, F, H, J) 25°C. (left) Emission spectra of hAK1 in the presence of statin, (right) dependence of fluorescence intensity on statin concentration. The concentration of hAK1 was 2 μM. The concentrations of five statins from a to h were 0, 2, 4, 8, 16, 24, 32, and 40 μM, respectively.

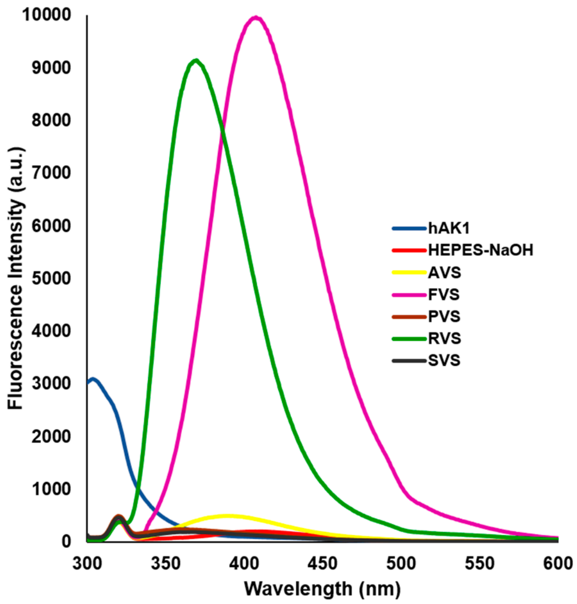

Figure S3. Emission spectra of 2 μM hAK1, 25 mM HEPES-NaOH (pH = 7.5), and five statins at the concentration of 40 μM.

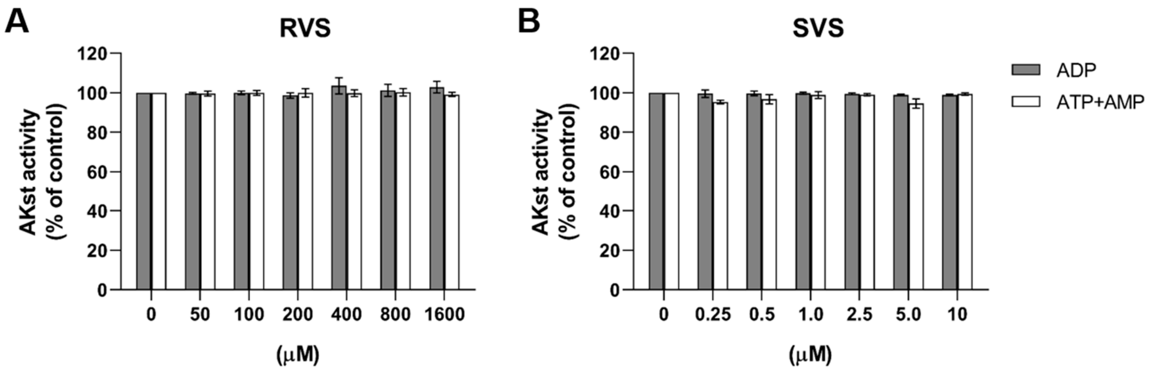

Figure S4. Effect of (A) rosuvastatin and (B) simvastatin on the AKst enzymatic activity in the presence of ADP or ATP and AMP as substrates, 1 mM each.

© 2021 by the authors. Submitted for possible open access publication under the terms and conditions of the Creative Commons Attribution (CC BY) license (http://creativecommons.org/licenses/by/4.0/).
